# Post-stroke aphasia rehabilitation using an adapted visual P300 brain-computer interface training: improvement over time, but specificity remains undetermined

**DOI:** 10.3389/fnhum.2024.1400336

**Published:** 2024-05-30

**Authors:** Sonja C. Kleih, Loic Botrel

**Affiliations:** Institute of Psychology, Biological Psychology, Clinical Psychology and Psychotherapy, Faculty of Human Sciences, Julius-Maximilians-Universität Würzburg, Würzburg, Germany

**Keywords:** brain-computer interface (BCI), aphasia, stroke, rehabilitation, P300 – event related potential, quality of life

## Abstract

**Introduction:**

This study aimed to evaluate the efficacy of visual P300 brain-computer interface use to support rehabilitation of chronic language production deficits commonly experienced by individuals with a left-sided stroke resulting in post-stroke aphasia.

**Methods:**

The study involved twelve participants, but five dropped out. Additionally, data points were missing for three participants in the remaining sample of seven participants. The participants underwent four assessments—a baseline, pre-assessment, post-assessment, and follow-up assessment. Between the pre-and post-assessment, the participants underwent at least 14 sessions of visual spelling using a brain-computer interface. The study aimed to investigate the impact of this intervention on attention, language production, and language comprehension and to determine whether there were any potential effects on quality of life and well-being.

**Results:**

None of the participants showed a consistent improvement in attention. All participants showed an improvement in spontaneous speech production, and three participants experienced a reduction in aphasia severity. We found an improvement in subjective quality of life and daily functioning. However, we cannot rule out the possibility of unspecific effects causing or at least contributing to these results.

**Conclusion:**

Due to challenges in assessing the patient population, resulting in a small sample size and missing data points, the results of using visual P300 brain-computer interfaces for chronic post-stroke aphasia rehabilitation are preliminary. Thus, we cannot decisively judge the potential of this approach.

## Introduction

Stroke is one of the leading causes of death and disability worldwide (Feigin et al., 2022). Regrettably, aphasia is diagnosed in 21 to 38% of stroke survivors (Berthier, 2005). Aphasia is a condition that hinders individuals to express themselves and comprehend language, severely limiting their ability to communicate and interact socially (Manning et al., 2021). In cases of motor aphasia, lesions in the Broca area (the pars opercularis and parts of the pars triangularis and the inferior frontal gyrus), the insula, the motor cortex, subcortical and periventricular regions were described (Ardila, 2010). Also, lesions in the parietooccipital region and basal ganglia involvement were reported (Bohra et al., 2015). When individuals suffer from motor aphasia, the activation of neurons in the Broca area and surrounding regions is hindered. As a result, the basal ganglia, cerebellum, thalamus, and precentral gyrus cannot be stimulated, which would in turn, activate brain stem nuclei responsible for the necessary muscle stimulation needed for language production (Hertrich et al., 2020). Insufficient communication ability can have adverse psychological effects, including depression and social withdrawal (Simmons-Mackie and Damico, 2007; Spaccavento et al., 2013). Speech therapy is often used as part of rehabilitation to prevent negative consequences and has been proven effective (Stephens, 2017). Unfortunately, aphasia symptoms persist and become chronic in approximately one-third of patients (Bakheit et al., 2007). Individuals with chronic aphasia often have limited treatment options, which can negatively impact their quality of life, affecting their social activity. Increased responsibility of caregivers may decrease patient self-efficacy (Spaccavento et al., 2013).

One way to express thoughts without muscular involvement is by using brain-computer interfaces (BCI) (Moore Jackson and Mappus, 2010). Using electroencephalography (EEG), brain activation can be detected and translated into a command controlling a connected device. In the case of the so-called P300 speller (Farwell and Donchin, 1988), a matrix containing the letters of the alphabet is presented to the user. Based on the oddball paradigm (Sutton et al., 1965), rows and columns are highlighted randomly. A P300 is elicited each time a target letter is highlighted as a reaction to the participant focusing his or her attention on this target letter of the matrix. A P300 is an event-related potential, occurring approximately 300 ms after the onset of a deviant stimulus, and shows a positive deflection of usually several μVolts (Pritchard, 1981). The BCI system can detect the letter the user focused on by calculating the one location in the matrix where a P300 occurred in the row and the column and presents this identified letter on a computer screen. Therefore, individuals with motor aphasia could use such a BCI system for communication if the stroke does not affect their language understanding (Shih et al., 2014).

However, due to the overlap of the brain regions involved in P300 generation (Polich, 2007) and motor aphasia, using a P300 BCI might also benefit aphasia rehabilitation (Kleih et al., 2016). It was assumed that generators of the P300 component are located in the prefrontal and temporoparietal regions (Polich, 2007). Therefore, frontoparietal integrity should lead to higher or more pronounced P300 amplitudes, and as the frontoparietal lobe is part of the language network (Hertrich et al., 2020), improving its integrity might also have a beneficial effect on symptoms of aphasia. To achieve such an effect, regular use of a BCI based on the P300 might be an option. In fact, it was shown that the P300 amplitude increases with training (Baykara et al., 2016) and with aphasia recovery (Nolfe et al., 2006). As the P300 amplitude is also determined by the attention level (Johnson, 1986), training P300 BCI spelling might support aphasia rehabilitation indirectly by improving attention (Arvaneh et al., 2019). There seems to be a neural overlap between attention and post-stroke aphasia. As reported by Varkanitsa et al. (2023), decreased connectivity in the dorsal attention network seems to be related to the severity of aphasia symptoms, and increased connectivity is a result of aphasia treatment. However, today, it is widely accepted that no single brain area is linked to one specific brain function only; rather, neural connectivity is responsible for intact brain functioning (Herbet and Duffau, 2020). In line with this assumption, it was shown that aphasia rehabilitation using word stimuli led to increased connectivity in language networks (Kiran et al., 2015).

In an earlier feasibility study on using P300 BCI for rehabilitation (Kleih et al., 2016), we found that people with post-stroke motor aphasia could learn to use a visual P300 BCI device. However, several questions remained unanswered in this study. We found a P300 amplitude increase throughout the training in some participants, but it remained unclear if this increase correlated with aphasia improvement. Additionally, we proposed that attention, particularly activation in prefrontal and frontoparietal regions, play a vital role in aphasia recovery. Unfortunately, our pilot study data set was insufficient to investigate this hypothesis. Although some participants were already in a chronic state, we could not rule out the effects of other rehabilitation treatments since all participants were enrolled in a rehabilitation schedule at the time of their assessment (Kleih et al., 2016).

Therefore, our objective for this study was to address the unresolved inquiries and introduce a dependable study plan to explore whether a visual P300 BCI therapy can enhance post-stroke motor aphasia. Our first hypothesis was that P300 BCI training would increase attention. We hypothesized this increase in attention to be measurable on a behavioral and a physiological level. Hypothesis 1a was, therefore, that test scores in neuropsychological attention tests improved between t1 and t2. Hypothesis 1b stated that the P300 amplitudes increased with P300 BCI training. Hypothesis 1c indicated that frontoparietal connectivity increased between t1 and t2. Our second hypothesis was that language production improved after the intervention compared to before. The third hypothesis was that psychological well-being and quality of life in patients with post-stroke motor aphasia improved after the P300 BCI training due to better language production abilities. Finally, with this study, we aimed to explore the influence of subjectively reported motivation, emotion, and attention on BCI performance and P300 amplitude. As prior research has identified a relationship between motivation, emotion, and brain signals used for BCI control (Kleih et al., 2010; Kleih-Dahms et al., 2021), we sought to understand this connection further.

## Methods

### Design

**Figure 1:**
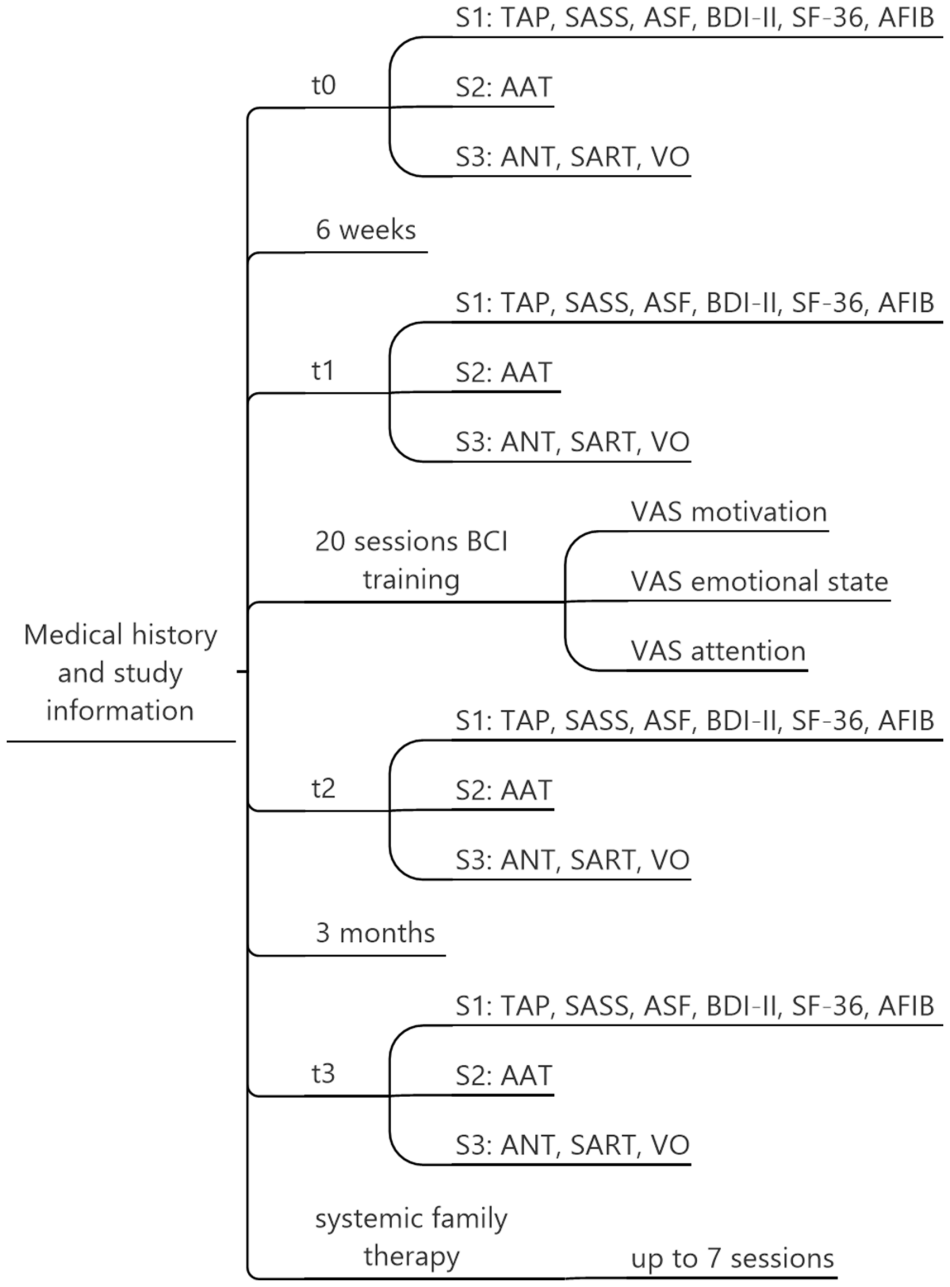
Assessment overview. Each participant underwent a total of 32 sessions: t0, t1, t2, and t3, with three sessions each (S1, S2, S3) and 20 sessions of BCI training. Based on individual needs, up to 7 additional therapy sessions were offered. TAP, Test Battery for Attention Performance; SASS, Social Activity Self-rating Scale; ASF, Aachen Self-efficiency Questionnaire; BDI-II, Beck Depression Inventory-II; SF-36, Short-Form-36 Health Survey; AFIB, Aachen Functioning Item Bank; AAT, Aachen Aphasia Test; ANT, Attention Network Task; SART, Sustained Attention to Reaction Task; VO, Visual Oddball Task.

We conducted a within-subjects study consisting of four assessments: baseline (t0), pre-test (t1), post-test (t2), and follow-up after three months (t3). The baseline was used as a control for extraneous factors independent of our intervention that could have affected our dependent variables. We hypothesized no changes between t0 and t1, a marked improvement in attention, language production and comprehension, and quality of life between t1 and t2, and we examined the durability of any observed changes over the long term by comparing t2 and t3. Each assessment included three sessions: S1, S2, and S3 (see Figure 1); therefore, we needed 12 sessions (4 × 3) overall to cover t0, t1, t2, and t3. In each S1 session, we assessed computerized attention tests and psychological questionnaires; in S2, we assessed the Aachen Aphasia test as a language production and comprehension instrument; and in S3, we assessed EEG-based attention tests (see section Instruments). The duration of S1, S2, and S3 sessions ranged from two to three hours each. Between t1 and t2, 20 P300 BCI training sessions were scheduled. Additionally, some participants received systemic family therapy sessions to address conflicts with family members that had arisen due to the stroke. These sessions were only conducted after the last data collection and are not part of this study or any other publication.

We initially planned to include at least *N* = 24 participants as this number would have been sufficient to detect a medium effect with four assessments, α=0.05, and β = 0.80 (G*Power Software, Faul et al., 2007). Participants were informed orally and written about the purposes of this study. The Institute of Psychology Ethics Committee from the University of Würzburg, Würzburg, Germany, evaluated and accepted the study.

### Participants

Inclusion criteria were symptoms of chronic motor aphasia after a one-time left hemispheric stroke, the ability to understand instructions, intact hand and wrist movement (for the button-press tasks), and the ability to visit our laboratory for participation. Exclusion criteria were several stroke events, a non-left-sided focus of the lesion, inability to visit the laboratory, cognitive impairment that prevented instruction understanding, impaired hand or wrist movement, epileptic seizures, or a pending pension request. All the individuals who participated in our study did not require a Brain-Computer Interface (BCI) system for communication purposes. A basic form of communication was preserved, such as using vocals or intonations and single words. They could communicate using non-verbal cues such as gestures, or facial expressions. If they had needed a communication device, a solution based on muscle activity would have been a better option for them regarding communication speed as compared to a BCI system (Lugo et al., 2015). Due to the COVID-19 pandemic, only twelve participants were enrolled. Five individuals terminated their participation early. Two withdrew due to medical reasons; two others felt overwhelmed with the intense training schedule on top of their hectic routine. For one, it was too time-consuming for the patient’s relatives to provide transportation to and from the training. Therefore, a sample of *N* = 7 participants remained (see Table 1). Participants were all male and *n* = 5 were retired because of chronic aphasia. In the remaining sample, we could collect data from all assessments (t0 to t3) in four participants; however, one finished 15 BCI sessions instead of 20. In patient B we could collect t0 data for the language production test but not for the other tests as he refused further tests before starting the P300 BCI training. In patient D, we assessed t0, t1, t2, and 14 sessions of P300 BCI training. We assessed data for t0 and t1 in patient E, and he participated in 12 BCI sessions. For him, participation ended due to a necessary in-patient treatment. In his initial report, a left-sided stroke was reported; in the later submitted report, a bilateral stroke event was reported (second stroke event). Medical information was accessed during the medical history assessment via the medical reports provided by the participants.

**Table 1:** Participants.

Participant	Age	Affected vascular territories	Years since the stroke event	No BCI sessions	Assessments
A	69	Middle cerebral artery	3	20	t0, t1, t2, t3
B	77	Middle cerebral artery	2	20	t0 (only AAT) t1, t2, t3
C	49	Middle cerebral artery	4	20	t0, t1, t2, t3
D	47	Middle cerebral artery	4	14	t0, t1, t2
E	52	Middle cerebral artery, posterior cerebral artery	3	12	t0, t1
F	52	Middle cerebral artery	8	20	t0, t1, t2, t3
G	56	Middle cerebral artery	6	15	t0, t1, t2, t3

### Instruments

#### Test battery for attention performance (TAP)

As a neuropsychological assessment of attention, we used the test battery for attention performance (Zimmermann and Fimm, 2012). We used the Alertness, Selective Attention and Sustained Attention subtests. According to Zimmermann and Fimm (2012), re-test reliabilities were reported to be in the range of 0.44 to 0.81.

The TAP Alertness test measures a person’s ability to respond to specific stimuli quickly and accurately. This test assesses a person’s overall state of alertness. Participants are presented with a cross at the center of the screen and must press a button as quickly as possible in response. A warning tone is presented before the button press during the second and third runs. The median reaction time was measured as the dependent variable in this test.

The Divided Attention subtest assesses whether an individual can simultaneously focus on equally important stimuli. Divided attention is considered a basic attention skill essential in everyday situations. In the TAP test, crosses are displayed randomly on a four-by-four matrix. Whenever four crosses form a square, a reaction button must be pressed. Simultaneously, two tones alternate, one with a higher pitch and one with a lower pitch. Whenever the same tone is repeated consecutively, a button must be pressed. The number of omissions is the most informative variable in this task.

The Sustained Attention test measures the ability to concentrate longer during a high mental load task. It assesses attention as required in a work environment. Various shapes with different sizes, colors, and fillings are shown during the test. Participants must press a button whenever a shape appears equal to the previously presented shape in at least two critical dimensions (consistent size, color, or filling). Errors and omissions, the dependent variables in this study, are crucial for interpretation.

#### Questionnaires

We used the Short Form-36 Health Survey (SF-36, Morfeld et al., 2011) to evaluate the quality of life based on subjectively perceived physical health. This tool consists of 36 items that measure different aspects of well-being, summarized in the subscales of physical function, role physical, role emotional, social function, pain, vitality, general health, and mental health. The responses are scored on a scale of 0 to 100, with higher values indicating better quality of life. Re-test reliability was on average 0.75.

Our study evaluated daily functioning using the Aachen Functioning Item Bank (AFIB) (Böcker et al., 2007). This tool measures daily functioning as an indicator of the quality of life, and we focused specifically on the cognition subscale, which comprises 18 items. The AFIB uses the Rasch transformation technique, and a score of 5.802 is the highest possible value, indicating optimal daily functioning.

We used the Aachen Self-Efficacy Scale (ASS, Wälte and Kröger, 1995) to measure self-efficacy. The scale comprises 20 items rated on a five-point Likert scale and assesses three domains: work/performance, interaction, and body/health. Higher scores indicate higher self-efficacy. According to the manual, raw values were transformed into percentile ranks. Re-test reliability was reported to range between 0.59 to 0.63.

We assessed social activity with the Social Activity Self-rating Scale (SASS, Duschek et al., 2003) which is a 20-item test that evaluates social functioning, motivation for social activity, and the quality of social relationships. The score ranges from 0 to 60 points, with higher scores indicating a higher level of social activity. Raw values are transformed to T norm values. Reliability was reported as 0.74.

We used the Beck Depression Inventory (BDI-II, Hautzinger et al., 2006) to evaluate the participants’ emotional state based on 21 questions. The scores can range from 0 to 63, with higher scores indicating higher severity of depressive symptoms. Re-test reliability was reported to be 0.93.

We used three visual analog scales to assess motivation, emotional state, and attention. All scales were 10 cm long lines with two extremes (highly motivated—not motivated at all, very good emotional state—very bad emotional state, and very attentive—not attentive at all). Participants indicated their motivational and emotional state and their level of attention by marking the position on the line that best corresponded to their subjective judgment. Visual analog scales were administered prior to every BCI session.

#### Aachen aphasia test

The Aachen Aphasia Test (AAT) was developed to assess expressive and receptive language abilities in individuals who have suffered brain injuries (Huber et al., 1983). It lasts between 60 and 90 min and comprises six subtests: Spontaneous Speech, Token Test, Repetition, Written Language, Denomination, and Speech Comprehension. Higher scores indicate higher functioning.

The Spontaneous Speech subtest assesses the ability to communicate, articulate, and follow the grammatically correct syntactic structure. A maximum of 30 points can be achieved. During the Token Test, the participant should organize colored shape platelets according to the instructions (for example: “touch the white circle after removing the yellow rectangle”). The complexity of the instruction increases over time and requires receptive language ability and correct execution. Repetition investigates expressive language ability as sounds, syllables, words, and sentences must be repeated and correctly pronounced. In the Written Language Test, language information must be transferred from the auditory modality to the tactile-motor ability. A dictation is performed. The Denomination test assesses the ability to identify objects correctly and perceive and describe situations accurately. In Speech Comprehension, the participant is asked to read a written word or sentence and choose the appropriate picture that shows this word or sentence as an image. T value norms are available for all subtests with the exception of the Spontaneous Speech test. Reliability was reported to be high, with a range between 0.86 and 0.97.

#### EEG-based tests

As a part of our study to measure attention on a brain activity level, we recorded EEG data while participants performed three tasks: the Attention Network Task (ANT; Fan et al., 2002), the Visual Oddball Task (VO), and the Sustained Attention to Response Task (SART; Robertson et al., 1997). For all these tasks, we built in-house versions using the software package Eprime^®^.

The ANT assesses alertness, orienting, and executive control. The participants were asked to perform eight blocks of five minutes and 32 trials each. Participants were given control to decide when to start the next block after completing one.

During a trial, a fixation cross appeared in the center of the screen. Above or below the cross, five arrows appeared. The participant’s task was to judge the direction in which the middle arrow pointed directly above or below the fixation cross. The other arrows were either congruent or incongruent with the target arrow. If the target arrow was pointed to the right, the letter L had to be pressed on the keyboard. If the target arrow pointed to the left, the A button had to be pressed. All other letter buttons were removed from the reaction keyboard to avoid unnecessary distraction. The difference in reaction times between the congruent and incongruent conditions indicated executive functioning. In half of the 256 trials, a small star served as a warning cue. The alerting efficiency was measured based on the difference in reaction times as a response to trials with and without the warning cue. In 50% of trials with a warning cue, it was presented on the top or bottom of the fixation cross, indicating the target location. In the other 50% of the trials, the warning cue appeared above and below the fixation cross, making it impossible to predict where the target stimulus would appear. The difference in reaction times between these two conditions represented the performance of the orienting reaction.

Participants were also presented with the visual oddball (VO) test. During the oddball task, 500 trials were presented over ten minutes. The letter ‘O’ was used to represent the deviant (oddball) target stimulus, while the letter ‘X’ was used for the repetitive standard stimulus. Participants were required to press the response key only when the letter ‘O’ appeared. The dependent variables were the reaction time, the P300 amplitude, and the number of correct responses.

During the SART task, participants were evaluated for their inhibitory control. A series of numbers ranging from one to nine were displayed randomly, with varying font styles and sizes on a screen. The participants were instructed to respond by pressing the spacebar on the keyboard after the presentation of numbers between zero to nine on the monitor except for the number three. The dependent variables were the reaction time and the number of errors.

### Data acquisition

During t0, t1, t2, t3 and the spelling sessions, participants were offered several minutes of rest between tasks. Participants were provided with written study information and gave their written informed consent.

#### TAP

Participants were seated 50 cm away from a 15-inch monitor for data acquisition using the TAP (Zimmermann and Fimm, 2012). The participants were instructed to rest both forearms on the table and the reaction keys were placed within easy and comfortable reach of their hands with the preferred finger above the reaction key. During the TAP tasks, the participants were instructed to react quickly by pressing the red dot on the reaction key. The participants were given written instructions on the screen, and the experimenter confirmed their comprehension. A pretest was carried out prior to the actual task to ensure that the task was understood and executed correctly.

#### EEG based tests

The ANT, VO, and SART were presented on a 15-inch monitor in an EEG cabin, with reaction keys consisting of two letters on the keyboard. For EEG measurement, we used 64 electrodes. The electrodes were FP1, FP2, AF7, AF3, AFz, AF4, AF8, F7, F5, F3, Fz, F2, F4, F6, F8, FT7, FC5, FC3, FC1, FCz, FC2, FC4, FC6, FT8, T7, C5, C3, C1, Cz, C2, C4, C6, T8, TP7, CP5, CP3, CP1, CPz, CP2, CP4, CP6, TP8, P7, P5, P3, P1, Pz, P2, P4, P6, P8, PO7, PO3, Poz, PO4, PO8, O1, Oz, and O2. Electrode FPz served as a ground electrode and the left earlobe as the reference. Three electrodes were used to measure the electrooculogram (EOG). Two of the electrodes were placed vertically above and below the right eye, while the third electrode was placed next to the right outer canthus to identify any horizontal eye movement artifacts. The impedances were at most 5 kΩ. We used a 64-channel amplifier (BrainAmp, Brain Products, Munich, Germany), with a sampling rate of 500 Hz, a high pass filter of 0.1 Hz, a low pass filter of 30 Hz, and a notch filter of 50 Hz.

##### P300 BCI sessions

We assessed data of 12 electrodes from the locations Fz, FCz, C3, Cz, C4, CPz, P3, Pz, P4, PO7, Oz, PO8. We used the right mastoid as a reference and the left as a ground electrode. Four electrodes assessed the electrooculogram (EOG). Two electrodes were placed vertically above and below the right eye, and two more were placed next to the outer canthi to identify horizontal eye movement artifacts. Impedances were at most 5 kΩ. We used a 16-channel amplifier (g.tec, Austria) with a sampling rate of 256 Hz, a high pass filter of 0.1 Hz, a low pass filter of 30 Hz, and a notch filter of 48–52 Hz.

For the P300 speller, a six-by-six matrix was used with the letters A to Z and several numbers. The matrix was presented on a 24-inch monitor, and participants were seated approximately 80 cm from the screen. The spelling process was controlled by the BCI 2000 software.^1^ We used ten sequences per letter, which equals 20 flashes to be counted mentally by the participant. On top of the matrix, the word to be spelled was written in a separate line. Right next to that word, the letter to be spelled during the ongoing trial was specified in parentheses. The participant had to locate this letter in the matrix and mentally count the times flashed. The two five-letter words BRAIN and POWER were used to calibrate the system using stepwise linear regression. During the copy-spelling, three five-letter words had to be spelled (RADIO, FUCHS, BLUME). During these copy-spelling runs, the letter recognized by the BCI system was presented in a separate line, so the participant received feedback about the spelling success. After detecting one letter with the BCI2000 system, the matrix remained static for 1 s to allow participants to locate the next letter. The duration of one flash was 62.5 ms, and the interstimulus interval was 250 ms. One trial equaled one letter choice and took 37.5 s. Participants were also offered a free-spelling mode in which they could freely spell words without copying predefined terms. The duration of one P300 session, including the electrode preparation, ranged between one and one and a half hours.

**Figure 2:**
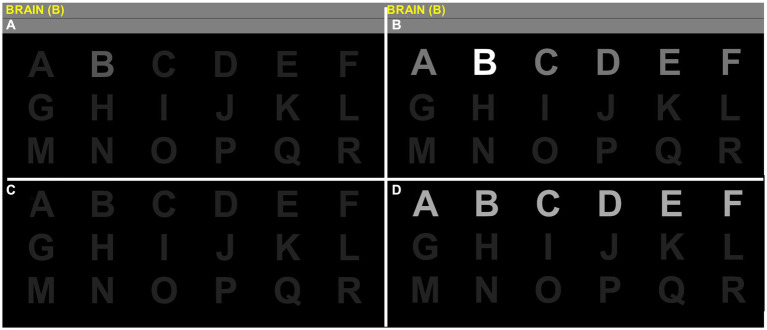
Spelling matrix in the facilitated version and the regular version. Stages of the visual P300 speller grid 6×6 containing letters A to Z and numbers 0 to 9 cropped in half for convenience. The cardboard condition is shown in the upper half (quadrants **A,B**), while quadrants **C,D** show a standard P300 display. The instruction is to spell the word BRAIN letter per letter, in this case, starting with the letter B. Participants always see the target inside the matrix (quadrant **A**) in the facilitated condition. During visual stimulation, the target is intensified stronger (quadrant **B**). Targets are not highlighted in the standard P300 setup (quadrant **C**) and do not stand out from other stimuli when highlighted during intensification (quadrant **D**).

To address the needs of our participants, we adjusted the training schedule individually according to the user-centered design (Kleih and Kübler, 2018) and the findings of our first study on this topic (Kleih et al., 2016). We reduced the planned number of three sessions per week to two sessions per week for two participants (E and F). Three participants (participants D, E and G) participated in in a reduced number of total sessions. Participant C was given new words (TANGO, ENZYM, and JUWEL) after session eleven due to reported boredom with spelling the same three words. All the participants were allowed to use a facilitated spelling paradigm during the experiment. In this paradigm, only the target letters were highlighted in white, while the rest of the stimuli were highlighted in light grey. The target stimulus was also presented in a lighter grey than the regular spelling matrix. In the regular spelling matrix, all stimuli were dark grey, and the entire row or column where the target stimulus was located was highlighted in light grey. For a visual representation, please refer to Figure 2.

During the P300 spelling task, the participants were required to spell the words presented and try to vocalize them aloud once they had completed spelling each word. This technique was implemented to increase the attention given to language production and to strengthen the expressive language ability component in this task, which is otherwise highly focused on comprehensive language processing. We asked participants to practice the spelling exercise mentally between t2 and t3.

### Data analysis

#### Instruments

The TAP, the questionnaires, and the AAT were analyzed using standardized values or raw values, as specified in the manuals.

#### P300 amplitude

Raw EEG data was re-referenced for the oddball sessions using common average reference (CAR), then bandpass filtered between 1 to 45 Hz. Data was then segmented into 800 ms epochs, and baseline corrected using 200 ms before the visual stimulus. Epochs were rejected if amplitude in the EEG channels exceeded a 100muV threshold. For each epoch that could either be deviant or repetitive, averages of C3 amplitude between 250 ms and 600 ms were collected to evaluate changes in P300 amplitude over time. Amplitude averages entered a linear mixed model (package lmerTest3.1–3) with the formula amplitude ~ session * stimulus + (1 + session * stimulus|subject) and post-hoc contrasts via means. For the P300 spelling, linear discriminant analysis was used as a classification method (Krusienski et al., 2008).

#### Behavioral indicators of attention in EEG-based tests

From the ANT test, we extracted (1) alerting by comparing trials with center cues to no cues; (2) orienting by comparing trials containing spatial information about the upcoming stimulus location compared to no cues, and (3) executive control by comparing incongruent stimuli to congruent stimuli. We extracted the number of omission errors, commission errors, and reaction time (RT) for each of these comparisons. For the SART test, we extracted (4) inhibition by comparing correct inhibition in response to NoGo stimuli as compared to Go stimuli with omission errors set at 1250 ms post-stimulus. For estimating omission and commission errors, we used a binomial (logit) generalized linear mixed-effect model (glmer from package lme4) with the formula DV ~ session + (1 + session|subject) and the same contrasts definition and post-hoc contrasts extraction method.

For the oddball paradigm, we extracted in a similar fashion (5) RT with omission errors set at 2166 ms after the stimulus. Omission errors were excluded from RT since no event occurred.

Right skewed reaction time could be assessed using robust linear mixed model regression (R package robustlmm3.3–1) with the following formula RT ~ session * comparison + (1 + session + comparison|subject) using pre-session as reference for a treatment contrast, with the comparison being between cues (i.e., alerting, orienting) or stimuli (i.e., executive control). Note that we could not use full random effects for the linear model to converge and had to simplify the model by removing the random interaction effect *subject: comparison*. Since the goal of the oddball task was to press only after deviant stimuli and such events occurred 20% of the time with a long 2-s inter-trial interval, there were not enough commission errors to perform a reaction time comparison, but we could compare the reaction time between sessions, which resulted in the following model: *RT ~ session + (1 + session|subject)*. Post-hoc treatment contrasts were calculated using estimated marginal means (R package emmeans1.9.0).

#### Cortical connectivity

Cortical connectivity investigated intra (density) and inter-cortical (influence) connectivity over time and comparisons. Connectivity estimates were extracted for every trial, every task, and frequency band of interest and then analyzed in LMM. For the ANT’s Alertness, orienting, and Executive control comparisons, as well as for the SART’s inhibition, we specifically focused on the interaction between time and comparison condition (e.g., deviant vs. repetitive in the case of executive function). For the P300’s Oddball, we only analyzed the effect of the factor time on connectivity estimates.

We used python3.9 data extraction and preprocessing and performed mne-python1.4.2. The inverse connectivity was performed using mne-connectivity0.5.0, and further statistics required statsmodels0.13.5.

For extracting data from the ANT task, we collected 400 ms post-cue onset for the components orienting and alerting and 500 ms post-stimulus onset for executive control. For computing the noise covariance matrix of the connectivity, we used the concatenation of all 500 ms windows before every cue onset. The SART trials had a cue-to-cue interval of 1.25 s from which we extracted 1 s post-cue. The noise covariance matrix was computed from the two-minute baseline acquired before each run.

The source reconstruction used the dynamic statistical parametric mapping (dSPM) method using FreeSurfer’s resources and libraries, notably “fsaverage-5120” as a reference brain model, and FreeSurfer’s “aparc” cortical parcellation. We used the “spectral_connectivity_time” function to compute the weighted phase lag index (wPLI, Vinck et al., 2011) specifying “cwt_morlet” mode (for wavelet method) to extract the connectivity in the frequency of interest with n_cycles = freqs/7. We separately extracted four EEG frequency bands of interest (i.e., theta, alpha, beta, gamma) and for multiple components (i.e., for ANT: alerting, orienting, and executive control; for SART: inhibition; for VO: all stimuli).

Every surface area lateralized for each hemisphere (e.g., orbitofrontal cortex-left, precuneus-right provided by the parcellation file), was calculated against another, resulting in a large matrix of wPLI estimates. The third dimension of the matrix represented every epoch (i.e., trial of the ANT conditions or of the SART test). Every surface area in these pairwise comparisons was distributed into two areas of interest. Those were prefrontal (20 areas) and parietal (8 areas) cortices (note that central labels: “paracentral” and “postcentral”) were left out. The frontal and parietal groupings allowed for the investigation of (1) cortical influence: the average connectivity between frontal and parietal areas; and the investigation of (2) cortical density (the average connectivity of within the group itself).

For estimating effects of influence between cortices and density within cortices, linear mixed models were used. We used the function MixedLM of the package stats models to integrate multiple within factors time (i.e., sessions pre and post) and comparison (e.g., for the alerting comparison, the difference between a center cue and no cue) as predictors for either cortical density or cortical influence, with full random-effects specified in the model. Prior to training the statistical models, the wPLI connectivity estimates were corrected by box-cox power transformation (from package scipy1.9.1) allowing for the linear mixed models to converge despite wPLI estimates being right-skewed. For the VO task, we did not compare between deviant and repetitive stimuli. Hence, the model was reduced to investigating connectivity estimates by the factor time with random time effects.

Based on previous literature (Anzolin et al., 2017), we focused on frontal–parietal influence. Specifically, we examined the alerting task by evaluating alpha activity. In the orienting task, we focused on the gamma band involving the left hemisphere. Lastly, we were interested in the beta band for the executive control task. Concerning connectivity, we did not have specific hypotheses for the VO task. Hence, we applied an alpha-level correction for multiple comparisons of *n* = 16 for density and *n* = 24 for influence.

## Data availability statement

The authors will share data upon reasonable request.

## Results

Due to our small sample size, we used robust or non-parametric tests for group-level analysis. To include participant D (i.e., the t3 session is missing) in the connectivity, reaction time, omission errors, and commission errors data, we used linear mixed models instead of mixed ANOVAs. Otherwise, to handle missing cases, we report descriptive data to interpret single cases. Overall, participants expressed that our research schedule was challenging. During the t0, t1, t2, and t3 sessions, they requested multiple breaks, ranging from five to 15 min. Participants A, C, and E reported sadness about their lost language abilities. We validated these feelings, but all participants continued data acquisition at their own pace.

### Attention

To test our first hypothesis that the attention level would increase due to the P300 BCI training, we analyzed the data of the TAP tests and the P300 amplitudes and investigated possible changes in connectivity as assessed with the ANT, SART, and VO tasks.

#### The TAP test as an indicator of attention

All the participants in the study scored below average in at least one attention domain on the TAP, which is in line with a subjectively reported perception of an attention deficit. During the test, most of the participants struggled with the sustained attention subtest and reported difficulty in staying alert. We present data for four participants (A, C, F, and G) for the baseline, t1, t2, and follow-up test series. For participants B, D, and E, their performance is reported only for the assessments where data is available (t0, t2, or t3). Using a Friedman ANOVA, we found no significant changes in alertness, neither without (*F*(3) = 3.40, *p* = 0.34) nor with a warning stimulus (*F*(3) = 0.32, *p* = 0.85). Participants A and B showed reaction times below average (< T = 40). In the divided attention subtest, we found no significant changes in omissions over time (*F*(3) = 1.29, *p* = 0.73). In this subtest, participants B, C and E showed T values below average.

The sustained attention subtest was performed only by two participants (C and G) for the whole test series due to exhaustion of the participants or difficulties in understanding the instruction. Therefore, a statistical analysis is impossible, and we report significant changes on a single case level according to the critical T value differences reported in the manual (Zimmermann and Fimm, 2012). All participants scored below average (T < 40) concerning the omissions. A significant improvement was only found in participant C for the follow-up assessment (t2 = 42 omissions vs. t3 = 13 omissions). Three participants also scored below average concerning errors, and we found a significant improvement in patient G between t2 (63 errors) and t3 (11 errors).

The performance of individuals in attention tests was found to be inconsistent, both between individuals and intraindividually. We did not observe any clear improvement in particular aspects of attention. Therefore, hypothesis 1a was rejected, which proposed that training with P300 BCI could enhance attention performance as measured by the TAP.

#### P300 amplitude as an indicator of attention

**Figure 3:**
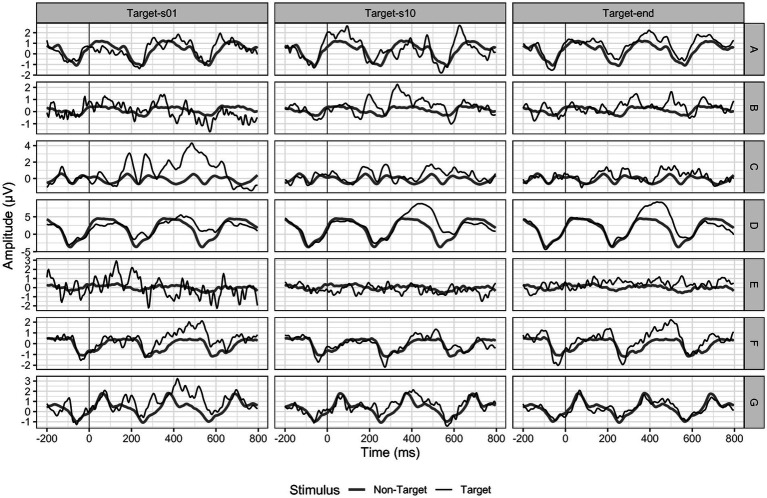
P300 amplitudes at electrode position Pz. Black = targets, dark grey = non-targets. Target end refers to session 20 for participants A, B, C, and F; to session 14 for participant D, to session 12 for participant E and to session 15 for participant G.

We conducted a non-parametric Friedman ANOVA to assess possible changes in the amplitude of P300 at a group level. Our analysis included data from seven participants for sessions one to 12. For sessions 13 and 14, we had data from six participants, while for session 15 we analyzed data from five participants. Finally, sessions 16 to 20 were completed by only four participants (see Figure 3). Participant E could not use the P300 speller without the cardboard paradigm version throughout his participation. He is the only participant who could not progress from the facilitated to the regular presentation after the first session. Two participants reported having continued practicing the spelling task mentally without a BCI device between t2 and t3.

Overall, the measured P300 amplitudes were very small and not comparable to the P300 deflection we would expect in a P300 BCI study with healthy adults. For electrode Fz, we found a non-significant decrease in P300 amplitude over time (*F*(19) = 26.19, *p* = 0.09). While the P300 was on average 0.77 μV (SD = 0.99) in session one, it decreased to 0.37 μV (SD = 0.80) in session ten and to 0.15 μV (SD = 0.30) in session 20. For electrode position Cz, we found a similar P300 decrease over time (*F*(19) = 19.97, *p* = 0.33). In session one, the mean P300 amplitude was 1.18 μV (SD = 1.30), while in session ten, it decreased to 0.68 μV (SD = 1.31) and to 0.34 (SD = 0.39) in session 20. For Pz (*F*(19) = 23.13, *p* = 0.19), we found a stable mean activity of 0.96 μV (SD = 1.09) between session one and ten (1.00 μV, SD = 1.40) and a decrease to session 20 of 0.60 μV (SD = 0.19).

As we assumed an increase in neuronal activation in the frontal–parietal area of the lesioned hemisphere, we also analyzed the P300 activation at electrode positions C3 and P3. In line with the results of the sagittal electrodes, we found a non-significant decrease of the P300 amplitude between session one and 20 (C3 *F*(19) = 19.16, *p* = 0.45; P3 *F*(19) = 12.61, *p* = 0.86).

When examining individual cases (see Figure 3), we observed a decrease in activation, contrary to our hypothesis of an increase, which supported the findings of the group analysis. Participant D is the only participant for whom we found a P300 amplitude increase over time, with an initial P300 at electrode position Pz of 2.55 μV in session one and 3.50 μV in session 20.

**Figure 4:**
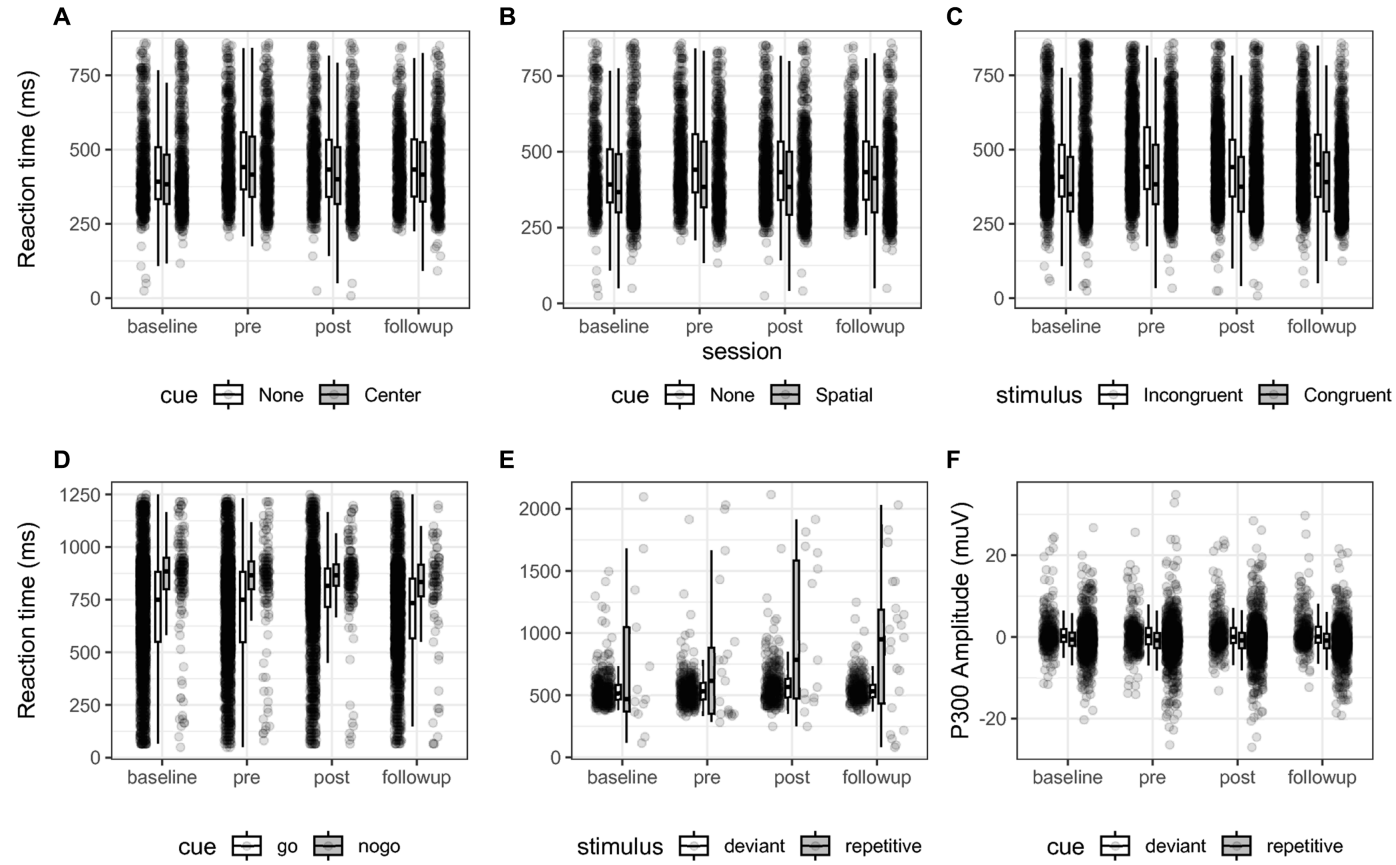
**(A–E)** Reaction time of all participants for each comparison of each behavioral test across sessions. Box-plots indicate quartiles, and whiskers extend until 1.50 interquartile range. **(A–C)** For the comparisons “Alerting”, “Orienting” and “Executive function” of the ANT test. **(D)** The “Inhibition” comparison of the SART test. **(E)** The “Oddball” comparison, reporting the reaction time press the button during deviant stimuli. **(F)** shows the P300 EEG amplitude between 250 to 600 ms post stimulus expressed in micro-Volts between deviant and repetitive cues. These plots illustrate the contrasts that were statistically evaluated between sessions. No significant interaction was found.

Another source of P300 amplitudes could be extracted from the visual oddball task. From the statistical LMER model we found no pre-post contrast between repetitive and deviant stimuli between 250 to 600 ms post stimulus (*t* (4.99) = 1.12, *p* = 0.31) for details see Figure 4F.

We rejected hypothesis 1b that the P300 amplitudes would increase over time.

#### Behavioral measures in the ANT, SART and VO as an indicator of attention

The ANT task measured executive control by the difference in reaction times between congruent and incongruent trials (see Figure 4). When averaging at the participant level, we found that incongruent cues descriptively delayed reaction time 53 ms at baseline, 60 ms at t1, 48 ms at t2, and 54 ms at follow-up, see Figure 4C. Post-hoc tests adjusted by Dunnett’s test for multiple comparisons returned no significant differences between reference level pre and levels baseline (*z* = 0.32, *p* = 0.96), post (*z* = −0.0004, *p* = 0.99) and follow-up (*z* = −0.17, *p* = 0.99) for the executive control comparison. *Post hoc* tests also evaluated the interaction of the comparison between sessions based on the binomial generalized mixed models. The *post hoc* tests showed that the odds of omission errors of incongruent stimuli as compared to congruent were reduced between baseline and pre at 0.22 the odds ratio CI (0.17, 0.30), z = −12.3, *p* < 0.001 and then increased between pre and post with 4.89 times the odds of omission errors CI (3.87, 6.17), *z* = 13.3, *p* < 0.001. Post-hoc contrasts showed that commission errors in executive control significantly decreased between pre and post representing 0.24 times the odds of CI (0.19, 0.32), *z* = −12.5, *p* < 0.001, and between pre and follow-up with 0.03 the odds CI (0.02, 0.05), *z* = −21.2, *p* < 0.001.

The alerting efficiency represents the difference between the response to trials with and without the warning cue. Descriptively, the presence of a central cue reduced reaction time by 3 ms at baseline, 23 ms at t1, 28 ms at t2 and 12 ms at follow-up. Post-hoc tests showed no significant differences in reaction time for the alerting comparison between measurements comparing the reference level pre and levels baseline (*z* = 0.29, *p* = 0.97), post (*z* = 0.03, *p* = 0.99) and follow-up (*z* = −0.19, *p* = 0.99). Post-hoc tests showed that by providing a central cue, the odds ratio of omission errors decreased with a ratio of 0.22 between baseline and pre, CI (0.15, 0.32), *z* = −9.52, *p* < 0.001 and increased with a 4.7 ratio between pre and post CI (3.3 6.8), *z* = 9.95, *p* < 0.001. The rate of commission errors due to the central cue decreased between pre and post with 0.25 the odds between CI (0.18, 0.36), *z* = −9.01, *p* < 0.001 and decreased with 0.03 the odds between pre and follow-up CI (0.02, 0.05), *z* = −16, *p* < 0.001.

To assess orienting, the difference in reaction time was measured between trials in which the warning stimulus indicated the position of the target stimulus and those without warnings. We observed this difference to be a reduction of descriptively 15 ms at baseline, 40 ms at t1, 39 ms at t2, and 28 at follow-up. Post-hoc tests yielded no significant difference in reaction time by introducing a directional cue between the reference level pre and levels baseline (*z* = 0.324, *p* = 0.96), post (*z* = 0.51, *p* = 0.99) and follow-up (*z* = −0.01, *p* = 0.99). In the post-hoc tests of the binomial generalized models, we found that the directional cue reduced omission errors between baseline and pre with a 0.23 the odds ratio CI (0.16, 0.33), *z* = −9.11, *p* < 0.001 and there was 4.6 times the odds of omission errors CI (3.2, 6.7), *z* = 9.55, *p* < 0.001 between pre and post. Commission errors decreased between pre and post in the orienting comparison with 0.24 times the odds CI (0.17, 0.35), *z* = −9.13, *p* < 0.001 and decreased between pre and follow-up with 0.03 the odds CI (0.02, 0.06), *z* = −15.7, *p* < 0.001.

The SART task measured response Inhibition, and we assessed the difference in reaction times between inhibition (i.e., NoGo, 20%) and press (i.e., Go, 80%) trials (see Figure 4D). When averaging all participants, we found reaction time to inhibition stimuli to be delayed 142 ms at baseline, 101 ms at t1, 106 ms at t2, and 84 ms at follow-up. Post-hoc tests did not reveal significant differences between sessions comparing the reference level pre and levels baseline (*z* = 0.07, *p* = 0.99), post (*z* = 1.28, *p* = 0.43) and follow-up (*z* = 0.586, *p* = 0.86). The post-hoc tests for the inhibition contrast showed that participants showed significantly more errors between baseline and pre with 0.80 the odds ratio of valid response CI (0.64, 0.99), *z* = −2.46, *p* = 0.04, did not significantly differ between pre and post (*z* = −2.07, *p* = 0.101), and then errors decreased between pre and follow-up with 1.3 the odds of valid response CI (1.1, 1.7), *z* = 2.97, *p* < 0.01.

For the VO task, we discriminated deviant stimuli and repetitive stimuli. Descriptively, we found global reaction times of 551 ms at baseline, 548 ms at t1, 560 ms at t2, and 552 ms at t3 (see Figure 4). Post-hoc tests for the oddball contrast revealed no difference in reaction time nor in P300 amplitude between sessions comparing the reference level pre and levels baseline (*z* = −0.31, *p* = 0.96), post (*z* = 2.05, *p* = 0.11) and follow-up (*z* = 1.27, *p* = 0.44). With commission and omission errors collapsed into valid and invalid trials, we found no significantly different odds for the oddball contrast between sessions comparing the reference level pre to levels baseline (*z* = 1.88, *p* = 0.15), post (*z* = −0.57, *p* = 0.86) and follow-up (*z* = −0.09, *p* = 0.99).

### Connectivity results

Connectivity was investigated both within the left frontal cortex (i.e., density) and for its connection between the left frontal cortex and other areas (i.e., influence). We compared between pre and post sessions and corrected *p*-values for hypothesis driven multiple comparisons. For the ANT alerting comparison, Linear mixed model contrasts showed no significant changes in connectivity in the alpha range from frontal-left to parietal left (*z* = −1.31 *p* = 0.38) and frontal-left to parietal-right (*z* = 0.26, *p* > 1) cortical areas. For the orienting comparison, we found no significant changes in the gamma band from left-hemispheric sources frontal-left to frontal-right (*z* = −0.95, *p* > 1), frontal-left to parietal-left (z = 0.05, p > 1), frontal-left to parietal-right (*z* = −0.06, *p* > 1), parietal-left to frontal-right (z = −1.46, *p* = 0.72), parietal-left to parietal-right (*z* = 0.38, *p* = 0.71). Density change was also non-significant in the frontal-left cortex (*z* = 0.31, *p* > 1) and the parietal-left cortex (*z* = −0.28, *p* > 1).

### Language production improvement

We conducted a statistical analysis using a Friedman ANOVA to evaluate the subtests of the AAT. We analyzed the data of patients A, B, C, F, and G, who completed all the required assessments. Our analysis revealed that symptom severity as, according to the AAT, improved in three participants. Two showed improvement between t1 and t2, while the other improved between t2 and t3. However, symptom severity remained the same for two participants throughout the study (refer to Table 2).

**Table 2:** Participants’ AAT raw scores and symptom severity for the baseline, t1, t2, and follow-up assessments.

	t0	t1	t2	t3
Participant A				
AAT SS	6	9	12	12
AAT TT	37	26	26	25
AAT RT	42	70	78	88
AAT WL	29	31	38	49
AAT DE	65	59	54	69
AAT LC	77	77	78	91
Symptom severity	Moderate	Moderate	Moderate	Moderate
Participant B				
AAT SS	19	22	22	25
AAT TT	7	7	3	0
AAT RT	137	137	147	148
AAT WL	87	79	88	88
AAT DE	111	110	116	116
AAT LC	106	95	96	106
Symptom severity	Mild/no aphasia	Mild/no aphasia	No aphasia	No aphasia
Participant C				
AAT SS	6	17	17	18
AAT TT	22	22	7	8
AAT RT	100	103	112	116
AAT WL	54	61	66	67
AAT DE	79	73	87	98
AAT LC	103	96	111	105
Symptom severity	Moderate/mild	Moderate/mild	Mild	Mild
Participant F				
AAT SS	4	9	10	12
AAT TT	19	7	16	16
AAT RT	70	102	104	111
AAT WL	19	28	23	37
AAT DE	20	55	52	26
AAT LC	76	85	98	92
Symptom severity	Moderate	Moderate	Mild	Moderate
Participant G				
AAT SS	24	27	28	29
AAT TT	15	8	4	9
AAT RT	137	142	133	143
AAT WL	85	82	81	87
AAT DE	97	93	101	112
AAT LC	103	100	101	117
Symptom severity	Mild	Mild	Mild	No aphasia

AAT, Aachen Aphasia Test; SS, Spontaneous Speech test; TT, Token test; RT, Repetition test; WL, Written language test; DE, Denomination test; LC, Language Comprehension test.

**Figure 5:**
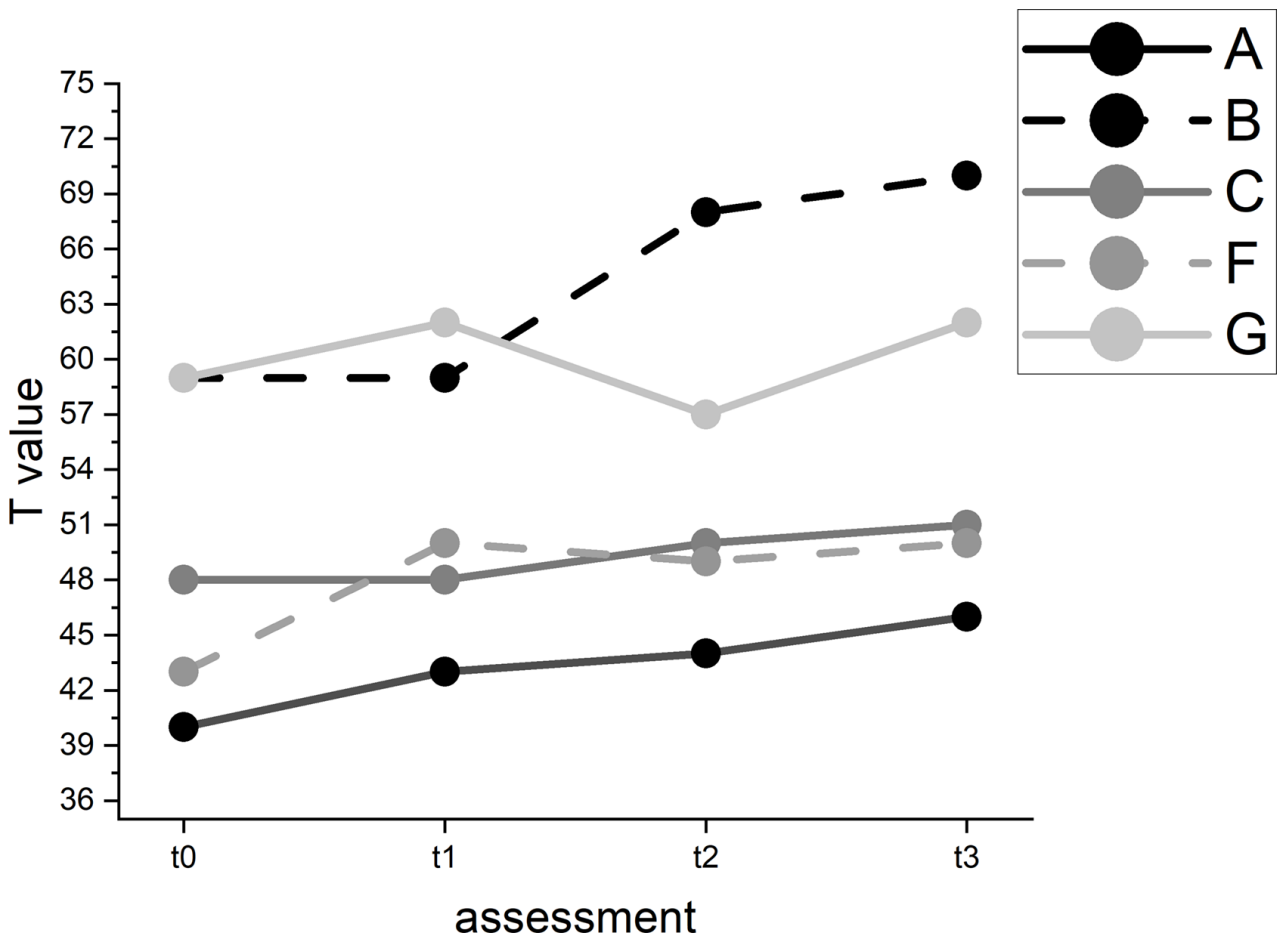
T value changes in the AAT repetition subtest for individual participants.

In the Token Test, we found no changes over time (*F*(3) = 7.51, *p* = 0.05). In the Repetition subtest, we found a significant improvement (*F*(3) = 9.52, *p* < 0.05) with a mean T value of 49.80 (SD = 8.87) at t0 and a mean T value of 55.80 (SD = 9.90) at follow-up (see Figure 5).

**Figure 6:**
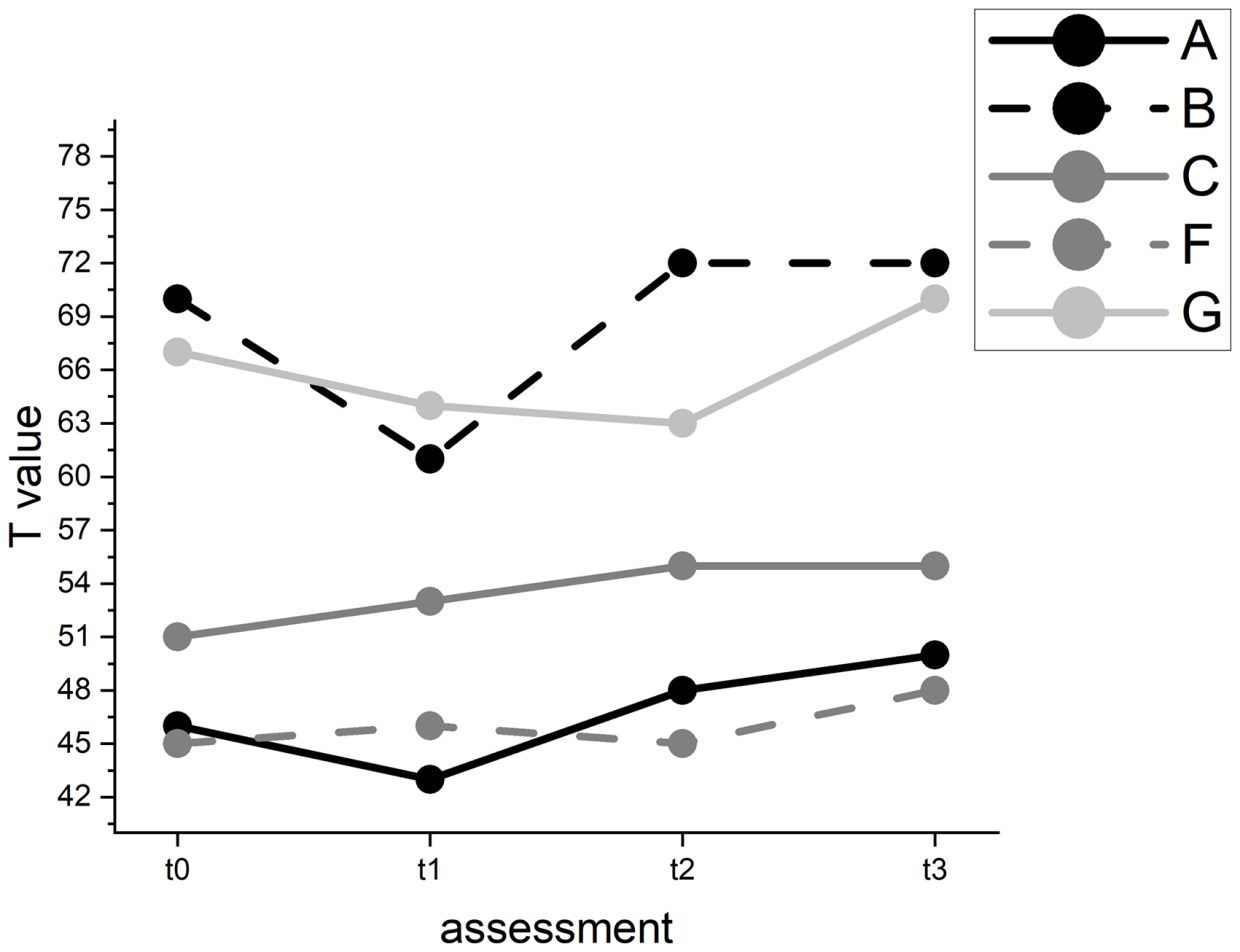
T value changes in the AAT Written Language test for individual participants.

However, pairwise comparisons revealed no significant improvement between t1 (*M* = 52.40, SD = 7.89) and t2 (*M* = 53.60, SD = 9.29). In the Written Language subtest, we found a significant improvement (*F*(3) = 8.11, *p* < 0.05) with a mean T value of 55.80 (SD = 11.86) at baseline and a mean T value of 59.00 (SD = 11.27) at follow-up (see Figure 6). Pairwise comparisons showed significant improvements between t0 and t3 and between t1 (*M* = 53.40, SD = 9.12) and t3 but not between t1 and t2 (*M* = 56.60, SD = 11.06).

In the Denomination subtest, we found no significant changes over time (*F*(3) = 6.32, *p* = 0.10). In Speech Comprehension we found a trend towards an improvement (*F*(3) = 7.78, *p* = 0.05) with a mean T value of 58.60 (SD = 8.88) at t0 and of 63.40 (SD = 9.44) at t3.

**Figure 7:**
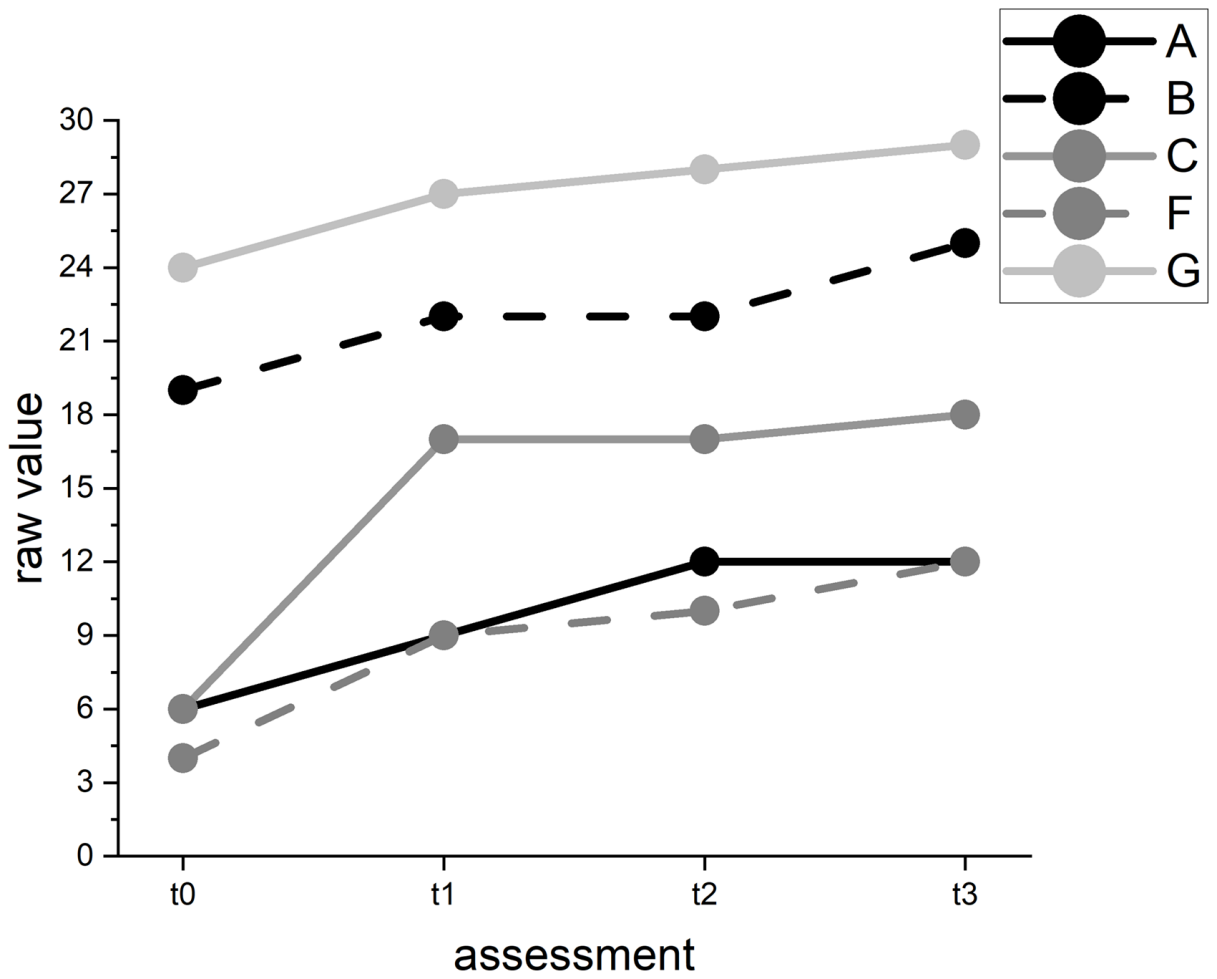
Raw value changes in the AAT Spontaneous Speech test for individual participants. Raw values range from 0 (worst) to 30 (best) points.

In the Spontaneous Speech test, we found improvement in all participants over time (*F*(3) = 14.23, *p* < 0.01). However, pairwise comparisons revealed an improvement between t0 and t2 and between t0 and follow-up but not between t1 and t2. On a descriptive case level, all participants improved between t1 and t2, and participants A and C even in several categories. However, all participants also improved between t0 and t1 (see Figure 7).

The results for our hypothesis that our training can improve language production and comprehension abilities were inconclusive. The most reliable improvement was found in the Spontaneous Speech subtest. However, we observed that in some subtests, the baseline (t0) test values were better than those at t1 but improved at t2 or t3. This pattern may represent fluctuations in language production and comprehension ability independent from our intervention. We rejected hypothesis 2.

### Psychological well-being and quality of life

Four patients (A, C, F, G) were analyzed for psychological well-being and quality of life using a Friedman ANOVA for repeated measures.

Our study revealed noteworthy findings with regards to the SF-36 questionnaire. We observed a significant improvement in the mental health sum score (*F*(3) = 8.10, *p* < 0.05), while the physical health sum score did not show significant changes (*F*(3) = 1.50, *p* = 0.68). Further pairwise comparisons revealed that there was a significant improvement between t1 (*M* = 41.81, SD = 11.48) and t3 (*M* = 80.00, SD = 10.19); however, we did not observe significant changes between t1 and t2 (*M* = 42.99, SD = 19.58).

Moreover, we found a significant improvement in subjectively experienced daily functioning, as assessed with the AFIB questionnaire (*F*(3) = 8.10, *p* < 0.05). Our pairwise comparisons revealed a significant improvement between t1 (*M* = 0.78, SD = 1.47) and t3 (*M* = 1.46, *SD* = 1.99), as well as between t2 (*M* = 0.63, SD = 1.07) and t3. For self-efficacy (ASS; F(3) = 4.71, *p* = 0.19), sociability (SASS; *F*(3) = 2.08, *p* = 0.56), and mood (BDI-II; *F*(3) = 5.92, *p* = 0.12), we found no significant changes over time. Across the test series, the mean self-efficacy scores ranged between 56 and 80 among the participants, the mean BDI scores ranged between 13 and 15, and the mean SASS scores ranged between 36 and 44.

The results do not unambiguously support our hypothesis that the P300 spelling training positively affects psychological well-being and quality of life by improving language production and comprehension. Between t2 and t3, these variables seemed to have improved, but we cannot rule out the possibility that unspecific effects contributed to these improvements rather than the P300 spelling practice alone.

### The influence of subjectively reported motivation, emotion, and attention on BCI performance and P300 amplitude

There was no consistent pattern of correlation between individual values for VAS motivation, emotion, attention and BCI performance, based on Spearman’s rho correlation when analyzing the data of participants A, B, C, F, and G. We found a single significant positive correlation between VAS attention and BCI performance in participant F (*r* = 0.73, *p* < 0.001). Average motivation was 7.64 (SD = 2.31) for all participants and over all sessions. The average emotional state was 7.86 (SD = 2.19), and the average attention was 7.62 (SD = 2.38). Interestingly, we noted that in three participants (A, F, and G), performances in percent for words they could choose themselves were higher with a smaller standard deviation compared to performances for the standard words and according standard deviations (see Table 3). For P300 amplitudes, we also only found one significant correlation between subjectively reported attention in the VAS and the P300 amplitude on Cz in participant G (*r* = 0.54, *p* < 0.05). In summary, we found no consistent significant relationship between motivation, emotional state, attention, and performance or P300 amplitude.

**Table 3:** Mean BCI performance in percent (*M*) for standard words and self-chosen words.

Participant	*M* standard word (SD)	*M* self-chosen word (SD)
A	59.65 (29.10)	65.67 (7.33)
B	80.66 (20.11)	73.87 (25.67)
C	82.98 (19.16)	76.75 (28.69)
F	70.30 (18.92)	83.30 (16.70)
G	79.97 (19.17)	87.88 (8.93)

*M* = mean, SD = standard deviation.

## Discussion

We investigated the potential of a visual P300 BCI for aphasia rehabilitation in stroke patients as a follow-up study to the 2016 feasibility study (Kleih et al., 2016). Despite implementing a rigorous research design, we cannot make a conclusive judgment on the usefulness of our intervention for post-stroke aphasia rehabilitation, mostly because of the small sample size and the missing control group. While we found improvements in the severity of aphasia in three patients, we cannot judge whether our intervention or other non-specific effects caused improvements. Due to high variability in assessed variables, we cannot establish a causal relationship between our intervention and improvements of dependent variables.

From our data, we could not measure a connection between attention, visual P300 amplitude, and aphasia rehabilitation. We found no increase in the neuropsychological test values for attention assessment, the P300 amplitudes, or the connectivity tasks. It might be that a potential link between the attention network (Varkanitsa et al., 2023) and the language network (Hertrich et al., 2020) cannot be influenced or measured by using a visual P300 BCI while with an auditory P300 BCI, a beneficial effect on post-stroke aphasia was suggested (Musso et al., 2022). Furthermore, the P300 amplitudes in this study were not comparable to the P300 amplitudes found in healthy participants (Dejanović et al., 2015). The reaction to deviant target stimuli was, in some cases, merely distinguishable from the reaction to repetitive stimuli, even though there is evidence that people after a stroke can show clearly pronounced P300 amplitudes (Dejanović et al., 2015). Successful use of a visual P300 BCI was shown in people who had a stroke and were diagnosed with aphasia (Shih et al., 2014). Our previous study also found successful P300 BCI use in a similar population (Kleih et al., 2016). However, the participants we included in this study were diagnosed with chronic aphasia symptoms, and all other treatment options were exhausted in this population. Therefore, the time window close to the stroke in which the highest neural plasticity can be expected (Nolfe et al., 2006) had long passed.

Even though we cannot judge the potential benefit of our intervention in improving language comprehension and production abilities, quality of life and the judgments of activities of daily living improved in our participants. This is in line with participants’ reports that through their study enrolment, they felt encouraged to engage in social interaction despite their aphasia symptoms, for example, in local associations where they had not dared to participate in years. As pointed out by Gabriel and Bowling (2004), one aspect of quality of life for older people is the feeling of retaining a role in society. Our participants understood that in this research, they could only contribute because they experienced a stroke and not despite having had a stroke. Some participants reported feelings of pride and joy from participating in this study. This feeling of contributing to society was found to be beneficial in the adaptation process of post-stroke aphasia (Manning et al., 2019). In that case, improvements in quality of life would have to be attributed to unspecific effects. Depending on the intervention, unspecific effects might occur (Kober et al., 2017). Kober et al. (2017) found specific effects for sensorimotor rhythm neurofeedback training to improve memory functioning, while only unspecific effects were found for neurofeedback training with the gamma band. On the other hand, a potentially hindering effect of a placebo on the ability for self-regulation in a neurofeedback paradigm was reported (Kober et al., 2018). However, in our sample, unspecific effects would most likely be attributed to psychological factors such as getting to the laboratory and interacting with the researcher, thereby being forced to communicate with a non-familiar person, which usually would be avoided (Harmon, 2020). Cognitive coping mechanisms, such as perceiving challenges as opportunities for personal growth, were reported to be effective in dealing with daily communication situations (Harmon, 2020). Therefore, by participating in our study, which involved interacting with unfamiliar people, our participants may have gained the confidence to communicate with other non-familiar individuals. Then again, we also faced a high number of dropouts. It may seem overwhelming for participants with post-stroke aphasia to perform 32 sessions. However, we needed to establish a baseline measurement to eliminate the possibility of exercise influencing dependent variables. Some participants in this study showed a drop in performance between the baseline and pre-intervention assessment and an increase between pre-and post-intervention assessment. These changes may not be considered natural performance fluctuations without the baseline assessment (Musso et al., 2022). The number of training sessions might, however, be reduced.

Although previous studies (Kleih et al., 2010; Kleih-Dahms et al., 2021) suggested a link between self-reported motivation and BCI performance and P300 amplitude, our study found no such connection. It is important to note that our sample size was small, and the P300 amplitudes we recorded were not comparable to those observed in healthy subjects using a visual P300 BCI (Halder et al., 2013). We could have analyzed the P300 in more detail or attempted to enhance classification accuracy by using machine learning algorithms (e.g., Fouad et al., 2020; Philip and George, 2020; Wang et al., 2021, 2023). However, our primary focus was to investigate using a P300 BCI for rehabilitation purposes, and the classification accuracies we found were quite high. Our results indicated that, on average, performance was better for self-chosen words than for standard words, suggesting that motivation may impact BCI performance beyond what a visual analog scale can measure.

Although we had ethical approval for our study, some may question whether it is ethical to invite post-stroke participants with aphasia to multiple laboratory sessions without being able to provide a treatment that has been proven to be beneficial, even though this is the case for every new treatment. In our information sessions, we were transparent with participants about the innovative nature of our research and the lack of proven results regarding treatment efficacy. Nonetheless, we noticed that many individuals interested in participating in the study were very hopeful and quite emotional. Some of the potential participants stated that they were hesitant to enroll unless they were given a guarantee that their aphasia symptoms would improve. We could not make any promises about potential symptom improvement, which led to one potential volunteer being discouraged from participating by a family member. These examples demonstrate that usual methods of informing participants may not be sufficient when working with participants who desperately wish to improve their situation for understandable reasons, as post-stroke aphasia has a tremendous impact on one’s personal life (Parr, 2007). In our research, we took care to avoid creating false hope (Musschenga, 2019) and made it clear that the treatment we used had not been tested before in this particular research design, and that we had only conducted a feasibility study (Kleih et al., 2016). Despite our care, one of our patients became angry and aggressive during the final session because he had expected to talk fluently again after participation. Another participant provided one medical report at the beginning of the study stating he had a left-sided single stroke event and met the inclusion criteria. After ten sessions, he handed us the second medical report, which stated he had had a second, bilateral stroke, which would have been an exclusion criterion. As it turned out, he could not continue participation anyway due to necessary medical treatment, but he knew that we would have excluded him in case he did not meet inclusion criteria. These experiences remind us of the vulnerability of our research clientele (Smith, 2008).

### Limitations

Our sample size is very small. This, and the fact that we did not assess a control group, limits the results of our research. Even though we planned to assess a control group initially who would have received biofeedback training instead of P300 BCI training, we could not follow through with this plan due to the pandemic. Also, methodologically, the number of participants we assessed in this study made it difficult to report the results of the data analysis optimally. We mainly opted for a group analysis despite the low number of participants. To include as much data as possible, we chose linear mixed models instead of ANOVAs for the behavioral analyses of the connectivity tasks, as those are robust to missing cases and could conveniently be extended to generalized linear mixed models for the binomial effects of commission and omission errors and provide post-hoc contrasts for specific comparisons. Linear mixed models added complexity due to the tradeoff when defining random effects, which we kept as maximally defined as possible and akin to repeated-measures ANOVAs (Matuschek et al., 2017).

The results provided by connectivity estimates between cortical surfaces returned large matrices. The usual procedure would be to calculate connectivity estimates over several epochs, however it would not provide enough samples to apply statistical methods. Hence we chose to extract connectivity on short individual epochs. The other difficulty we faced was the right skewed nature of the wPLI connectivity estimate, which would not allow the linear mixed model to converge properly. We used the box-cox method to transform our connectivity estimates non-linearly. We tackled the topographical complexity of connectivity sources by regrouping surfaces in lateralized frontal and parietal cortices, reducing the number of comparisons. The results were subject to a false discovery rate as we analyzed density and influence at delta, alpha, beta, and gamma ranges. Yet our intention was also not to miss potential effects, and so we constrained our analysis only to the replication of the findings in Anzolin et al. (2017), always including the frontal left cortex and in a specific frequency range, then Bonferroni adjusting the significance to the number of pairwise comparisons.

As mentioned before, we cannot exclude unspecific effects having caused or contributed to our findings. Data showed high intraindividual and interindividual variance, and improvements in spontaneous speech and perceived quality of life might have been caused by social engagement during participation (Manning et al., 2019). Furthermore, a strong selection bias must be assumed for our sample as we recruited participants from an advertisement in a local newspaper. Judging the recruitment procedure now, it might have been better to invite prospective participants via cooperating clinics or university hospitals as more participants could have been reached before the pandemic. Additionally, future studies should calculate a long-term time frame for such studies as the effort for participants, and researchers is tremendous, and only several-year projects with researchers also hired for such several-year periods can answer a research question like this.

We included participants with varying degrees of aphasia, resulting in high heterogeneity among the small participant group. Also, we found the best improvement in aphasia severity in the ones that were least affected from the beginning, which was shown before (Musso et al., 2022). This intervention was created for the patients for whom all other treatment options were exhausted, but it seems that we cannot answer whether those most affected can benefit from it.

## Conclusion

At this point, we cannot answer the question of whether visual P300 BCI spelling is a beneficial intervention method for the rehabilitation of post-stroke aphasia. Future research with more participants and a control group, recruited in cooperation with a clinic or university hospital, is necessary. Our results showed an improvement in aphasia severity and quality of life, of which we do not know whether it can be attributed to our intervention or unspecific effects. A long-term project with sufficient funds and personnel is required to tackle this question. Future researchers must be aware of the potential false hopes of participants and should try to mitigate the risk of supporting wrong beliefs about potential efficacy.

## Data availability statement

The raw data supporting the conclusions of this article will be made available by the authors upon reasonable request.

## Ethics statement

The studies involving humans were approved by Ethical Committee of the Psychological Institute of the University of Würzburg, Würzburg, Germany. The studies were conducted in accordance with the local legislation and institutional requirements. Written informed consent for participation in this study was given by the participants themselves.

## Author contributions

SK: Conceptualization, Data curation, Formal analysis, Investigation, Methodology, Project administration, Writing – original draft, Writing – review & editing. LB: Formal analysis, Investigation, Software, Visualization, Writing – review & editing.

## Footnotes

^1^ www.bci2000.org
